# Potential Biotechnological Applications of Autophagy for Agriculture

**DOI:** 10.3389/fpls.2021.760407

**Published:** 2021-10-27

**Authors:** Nipuni Thanthrige, Sudipta Das Bhowmik, Brett J. Ferguson, Mehdi Kabbage, Sagadevan G. Mundree, Brett Williams

**Affiliations:** ^1^Centre for Agriculture and the Bioeconomy, Queensland University of Technology, Brisbane, QLD, Australia; ^2^School of Agriculture and Food Sciences, University of Queensland, Brisbane, QLD, Australia; ^3^Department of Plant Pathology, University of Wisconsin-Madison, Madison, WI, United States

**Keywords:** autophagy, pathogen resistance, crop improvement, abiotic stress, senescence, programmed cell death, stress response

## Abstract

Autophagy is a genetically regulated, eukaryotic cellular degradation system that sequestrates cytoplasmic materials in specialised vesicles, termed autophagosomes, for delivery and breakdown in the lysosome or vacuole. In plants, autophagy plays essential roles in development (e.g., senescence) and responses to abiotic (e.g., nutrient starvation, drought and oxidative stress) and biotic stresses (e.g., hypersensitive response). Initially, autophagy was considered a non-selective bulk degradation mechanism that provides energy and building blocks for homeostatic balance during stress. Recent studies, however, reveal that autophagy may be more subtle and selectively target ubiquitylated protein aggregates, protein complexes and even organelles for degradation to regulate vital cellular processes even during favourable conditions. The selective nature of autophagy lends itself to potential manipulation and exploitation as part of designer protein turnover machinery for the development of stress-tolerant and disease-resistant crops, crops with increased yield potential and agricultural efficiency and reduced post-harvest losses. Here, we discuss our current understanding of autophagy and speculate its potential manipulation for improved agricultural performance.

## Introduction

Plants must intricately balance their energy needs for growth and development with survival and stress responses. Autophagy contributes to this balance by trafficking and degrading/recycling unwanted cytoplasmic materials in the vacuole (plants) or lysosome (mammals; Masclaux-Daubresse et al., 2017; Bozhkov, 2018; Yoshimoto and Ohsumi, 2018). With roles in cancer, ageing, diabetes and numerous neurodegenerative diseases, the identification of autophagy components and regulatory machinery is one of the most research-intensive fields in mammalian biology (Hayat, 2017). Likewise, autophagy is crucial for the proper regulation of plant metabolism and nutrient remobilisation in response to biotic and abiotic stress and a housekeeping capacity. With climate change models suggesting more frequent droughts and unpredictable weather patterns in the future, the importance of autophagy in plant stress responses is gaining significant momentum (Liu et al., 2009; Tang and Bassham, 2018; Gou et al., 2019). The dual role of non-selective and selective autophagy allows bulk or targeted degradation of protein complexes and organelles (Kellner et al., 2017; Wang et al., 2018). The manipulation of plant autophagy pathways for the development of stress-tolerant and disease-resistant crops with increased yields and agricultural efficiency, and minimal post-harvest losses, is now feasible and may play a significant role in sustaining agriculture in changing climates. Here, we discuss the roles of autophagy in plant stress biology and potential ways to manipulate the process for improved crop performance.

## Autophagy in Crop Stress Responses

The role of autophagy in response to plant stress is well-established (Kabbage et al., 2017; Avin-Wittenberg et al., 2018; Tang and Bassham, 2018; Avin-Wittenberg, 2019; Thanthrige et al., 2020). Stress directly damages proteins and membranes; it also causes the accumulation of unfolded proteins and endoplasmic reticulum (ER) stress that trigger programmed cell death (PCD) pathways. To avoid unwanted cell death, cells initiate corrective measures that remove misfolded or damaged proteins, including the unfolded protein response (UPR) and ER-associated degradation (ERAD) in a coordinated manner with the ubiquitin proteasome system (UPS) and autophagy (Li et al., 2020; Qu et al., 2021). Ubiquitin-mediated protein degradation occurs through proteasome or autophagy pathways (Su et al., 2020; Singh et al., 2021). While the UPS targets the degradation of ubiquitylated, short-lived, individual misfolded or regulatory polypeptides, autophagy eliminates individual misfolded proteins and bulk structures, such as large protein complexes, insoluble protein aggregates and organelles (Singh et al., 2021). Due to its specificity, the degradation of protein aggregates by the UPS is inefficient during prolonged stress (Toyooka et al., 2006). Therefore, autophagy represents a robust mechanism for the complete and efficient large-scale degradation of aggregated proteins during severe or prolonged stress conditions (Toyooka et al., 2006; Xiong et al., 2007). In the next few sections, we discuss how autophagy plays a role in plant stress responses.

Abiotic and biotic stresses impede electron transport chains and cause the accumulation of reactive oxygen species (ROS) that directly damage proteins, causing their partial denaturation and aggregation. Plant autophagy pathways are highly attuned to the oxidative state of the cell as part of housekeeping mechanisms to modulate ROS damage (Tang and Bassham, 2018). During stress and the accumulation of ROS, the oxidation of the autophagy gene, *ATG4*, triggers corrective autophagy pathways that mitigate cellular ROS levels by removing ROS generators, such as dysfunctional mitochondria or chloroplasts and removing oxidised proteins from the cell (Xiong et al., 2007; Tang and Bassham, 2018). In addition to ROS, many stresses, such as drought, cause the cessation of photosynthesis resulting in caloric and other nutritional deficiencies that also trigger autophagy pathways (Williams et al., 2015).

Drought and salinity induce the expression of autophagy genes (e.g., *ATG18a*), and Arabidopsis knockdown plants with defective autophagy pathways are sensitive to abiotic stresses (Liu et al., 2009). Autophagy’s efficient degradation of select proteins and large aggregates or organelles facilitates tolerance against severe stress states, such as desiccation (Williams et al., 2015; Oliver et al., 2020). Recent studies demonstrate that the native Australian resurrection grass, *Tripogon loliiformis*, uses trehalose metabolism to promote and maintain autophagy pathways that prevent senescence and PCD (Williams et al., 2015; Asami et al., 2019; Okemo et al., 2021). A similar role for autophagy in desiccation tolerance occurs for the resurrection plant, *Boea hygrometrica* (Zhu et al., 2015). Of particular note, the shoots and roots of *T. loliiformis* use different desiccation response strategies. *Tripogon loliiformis* shuts down photosynthesis during the early stages of drying and uses autophagy to transport resources from the shoots to the roots. The photosynthetic shutdown also slows down transpiration and water loss. While the remobilisation of resources helps *T. loliiformis* roots maintain energy homeostasis, mitigating the need to implement harsher survival measures, including autophagy (Asami et al., 2019). Whether a similar process occurs in Poikilochlorophyllous resurrection plants that remobilise their nitrogen sources *via* the degradation of chlorophyll remains to be seen confirmed (Costa et al., 2017; Asami et al., 2018).

Autophagy is a double-edged sword with survival and cell death roles in plant-pathogen interactions (Lenz et al., 2011; Thanthrige et al., 2020). Autophagy modulates defence responses regulated by salicylic acid (SA) and jasmonic acid (JA), thereby influencing plant basal resistance to both biotrophic and necrotrophic pathogens (Liao and Bassham, 2020). During necrotrophic fungal infection, autophagy positively regulates plant defences, serving an anti-death role to limit the disease lesion by suppressing ROS-mediated accumulation of oxidised compounds and subsequent lesion development and disease containment (Lenz et al., 2011). Autophagy-deficient mutants are susceptible to disease (Lai et al., 2011; Lenz et al., 2011). Conversely, the pro-death attributes of autophagy support host defence against viruses. In host plant-virus interactions, host-regulated autophagy kills surrounding uninfected cells, limiting disease spread (Hofius et al., 2009). The benefits of autophagy death for biotrophs in general are unclear (Leary et al., 2019).

Autophagy plays a role in plant responses to pathogens by positively regulating SA accumulation (Sun et al., 2018a,b). Overexpression of *ATG18a* in apple enhances SA levels and improve resistance to the fungal pathogen *Diplocarpon mali* (Sun et al., 2018a,b). The transcription factor WRKY33 interacts with *ATG18a* to regulate autophagy. The induction of *ATG18* and autophagy was reduced in *wrky33* mutants. Furthermore, autophagy mutants contained dysfunctional JA-mediated signalling pathways and were more susceptible to *Botrytis cinerea* (Lai et al., 2011). Similarly, autophagy defective banana are more susceptible to Fusarium wilt; the exogenous application of SA and JA can rescue the sensitive phenotype (Wei et al., 2017). The hypersensitive response (HR) is a localised form of PCD that occurs during plant-microbe interactions (Liu et al., 2005). Autophagy regulates the HR, and autophagy mutants display runaway cell death (Liu et al., 2005). Thus, autophagy plays an essential ‘pro-survival’ function in plants that effectively control the pathogen spread without resulting in ‘unwanted’ cell death in innocent uninfected bystander cells (Liu et al., 2005). Conversely, a pro-death function of autophagy during hypersensitive cell death has also been demonstrated (Hofius et al., 2009). In Arabidopsis, *atg* knockout mutants (*atg7* and *atg9*) display delayed HR PCD induced by *Pseudomonas syringae* pv. tomato DC3000 harbouring the avirulence genes *AvrRps4* (Hofius et al., 2009). Subsequent studies showed that Bax inhibitor-1 (BI-1) interacts with *ATG6* to positively regulate autophagy (Xu et al., 2017). Silencing of BI-1 reduces the autophagic response to TMV, whereas overexpression of BI-1 increased autophagic activity and enhanced defence to viral infection (Xu et al., 2017).

## Autophagy in Symbiotic ‘Friend Versus FOE’ Relationships

Autophagy plays a role in symbiotic relationships, including rhizobia-legume interactions. As an established inducer of autophagy, trehalose accumulates during symbiotic plant interactions (Brechenmacher et al., 2010; Williams et al., 2015; Li et al., 2016b). Silencing of trehalase, the enzyme that breaks down trehalose in common bean (*Phaseolus vulgaris* L.), increases trehalose content and supports augmented bacteroid number, nodule biomass and nitrogenase activity (Barraza et al., 2013). Transgenic *TRE1*-RNAi nodules accumulate more ATG3 transcripts, further suggesting a role for autophagy in nodule formation (Barraza et al., 2013). Studies on transgenic soybean expressing the *CED-9* from *Caenorhabditis elegans* display reduced nodulation and organogenesis, possibly by interfering with autophagy and vesicle trafficking (Robert et al., 2014). Similarly, studies in mammalian cells demonstrated an association between CED-9/Bcl-2 and Beclin-1/ATG6 to show a link between the anti-apoptotic activities of CED-9 and autophagy (Takacs-Vellai et al., 2005). However, further studies must be conducted to consolidate these links and what implications they have for plant autophagy.

## Autophagy in Nutrient Remobilisation and Plant Development

Autophagy and the UPS play pivotal roles in nutrient recycling and remobilisation and are distinguished by their capacity to function over distance (Wang et al., 2017; Avin-Wittenberg et al., 2018; Tang and Bassham, 2018). The UPS degrades individual proteins/protein aggregates and is not a suitable target for large-scale nutrient remobilisation. In optimal conditions, with low levels of aggregated proteins, the UPS mediates the majority of targeted protein degradation. By contrast, autophagy can function over the entire cell and degrade complete organelles. Plants often require rapid recycling, large-scale remobilisation and reabsorption of nutrients. Therefore, upon nutrient starvation and environmental stress, autophagy functions as a brute force yet efficient system to remobilise nutrients at scale (Doelling et al., 2002; Thompson et al., 2005).

### Manipulation of Autophagy for Improved Stress Tolerance

Plants are continuously exposed to multiple stressors in their environment and modulate their growth and development accordingly; autophagy plays comprehensive roles in many of these responses. Furthering our knowledge about autophagy roles in stress responses provides possible routes for new strategies and candidates for crop improvement, promoting stress resistance and yield.

**Figure 1:**
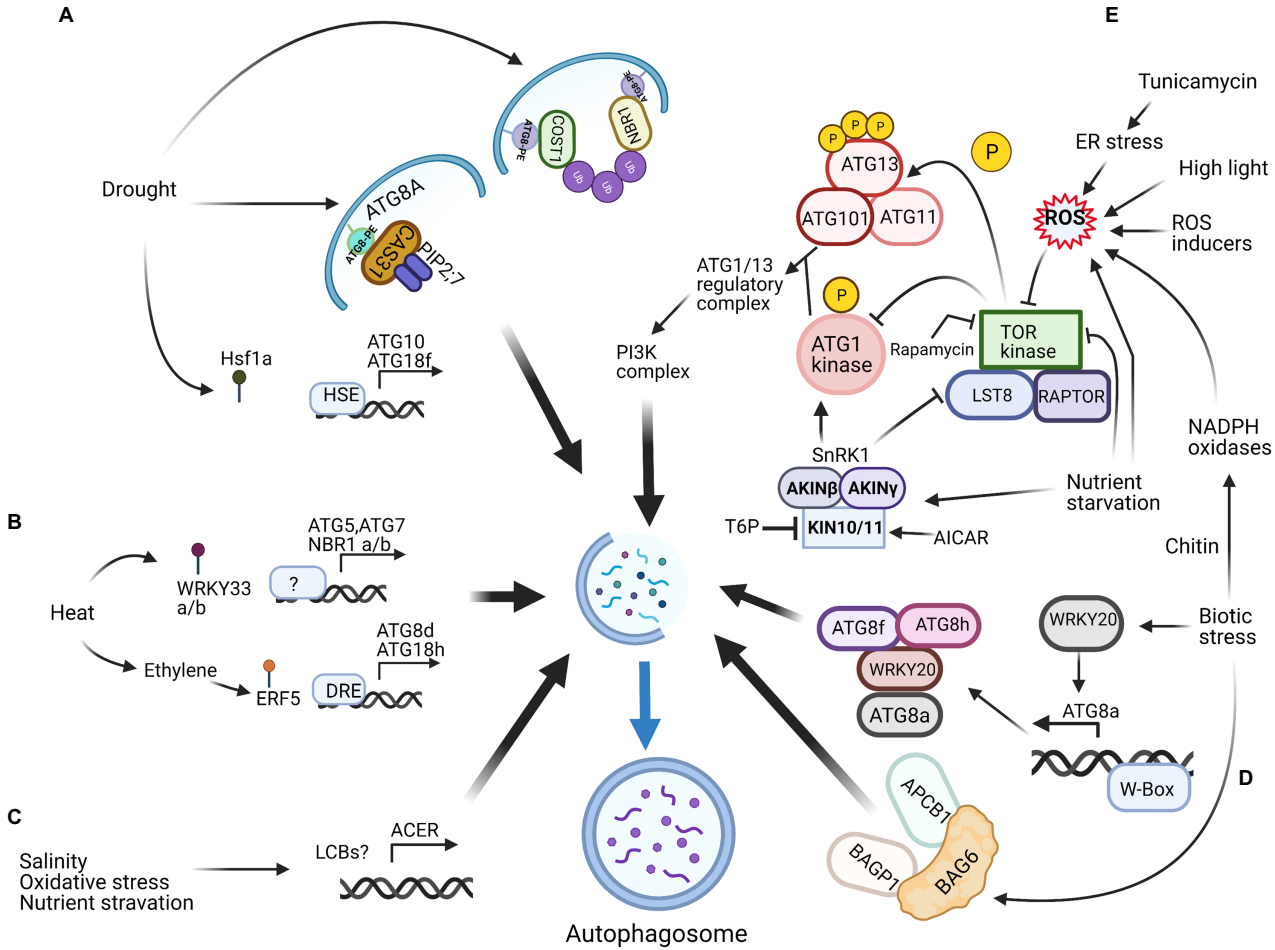
Potential targets for manipulation of autophagy in plants. Identified regulators and potential ATG gene targets during abiotic stresses such as drought stress **(A)**, heat stress **(B)**, salinity, oxidative and nutrient starvation stresses **(C)**, and biotic stresses **(D)** and stresses caused by reactive oxygen species **(E)**. Refer text for the detailed information. NBR1, Neighbour of BRCA1; COST1, Constitutively Stressed 1; HSE, Heat shock element; ATG, Autophagy related; PE, Phosphatidylethanolamine; PIPs, Plasma membrane intrinsic proteins; Ub, Ubiquitin; HsfA1a, Heat-shock transcription factor A1a; ERF5, Ethylene response factor 5; DRE, Drought-responsive elements; ACER, Alkaline ceramidase; LCBs, Long‐chain bases; PI3K, Phosphatidylinositol-3-phosphate kinase; SnRK1, Sucrose nonfermenting1-related protein kinase1; TOR, Target of rapamycin; T6P, Trehalose-6-phosphate; AICAR, 5-Aminoimidazole-4-carboxamide ribonucleoside monophosphate; BAGP1, BAG-Associated GRAM Protein; APCB1, Aspartyl protease cleaving BAG; ROS, Reactive oxygen species. This figure was created with Biorender.

Autophagy can be regulated genetically (i.e., genetically modified or edited crops) or pharmacologically (Avin-Wittenberg et al., 2018). Before use as a biotechnological strategy for improving crops, however, reliable systems for the precise manipulation of autophagy must be available. Next, we discuss the potential manipulation of autophagy pathways under different stress conditions and speculate on potential targets of plant autophagy machinery for crop improvement (Figure 1).

#### Overexpression of Autophagy Genes

There is significant potential to manipulate the regulation of autophagy pathways for improved crop tolerance to abiotic stresses. For example, drought activates autophagy pathways in crops and transgenic crops, such as apple, wheat, tomato, foxtail millet and barley, overexpressing *ATG* genes display significantly improved drought tolerance (Kuzuoglu-Ozturk et al., 2012; Pei et al., 2014; Li et al., 2015b, 2016a; Wang et al., 2015, 2017; Zeng et al., 2017; Mamun et al., 2018; Sun et al., 2018c). Notably, the expression of *ATG* genes in crops resulted in no detrimental effects in non-stressed conditions. In addition to *ATG*s, genes that modulate autophagy pathways are suitable targets for crop improvement. However, as opposed to the expression of *ATG* genes, a compromise between yield and tolerance must often be satisfied when expressing autophagy regulators to minimise detrimental effects and gain agronomical benefits.

As an inducer or a suppressor, the dual role of autophagy in biotic stress responses lends itself to the generation of pathogen resistance (Liu et al., 2005). Furthermore, and similar to abiotic stress responses, other than *ATG6*, the expression of autophagy genes does not have detrimental effects on plant growth and yield; thus, *ATG*s are a good target for developing resistant crops. Plants overexpressing autophagy genes (e.g., *ATG5* and *ATG7)* display improved stress tolerance and resistance against pathogens (Minina et al., 2018). A set of glyceraldehyde-3-phosphate dehydrogenases (*GAPDHs*) interact with ATG3 to negatively regulated disease resistance in *Arabidopsis*, *Nicotiana benthamiana* and cassava. Contrastingly, silencing of cytosolic glycolytic *GAPDHs* (*GAPCs*) enhanced disease resistance (Han et al., 2015; Henry et al., 2015; Zeng et al., 2018).

Another potential gene target for crop improvement is alkaline ceramidase (ACER), an essential enzyme in the sphingolipid metabolic pathway. Silencing of *ACER* inhibits autophagy. Conversely, its overexpression promotes autophagy under nutrient starvation, salinity and oxidative stresses (Zheng et al., 2018). Plant hormone signalling pathways are also targets for the regulation of autophagy. For example, the overexpression of *ERF5* (an ethylene response factor) improves drought tolerance in tomato (Pan et al., 2012). *ERF5* directly binds to the promoters of *ATG8d* and *ATG18h* and activates gene expression to promote autophagy, which is essential for ethylene-mediated drought resistance (Zhu et al., 2018; Figure 1).

#### Transcription Factors as Targets

Similar to drought, the modulation of autophagy regulators can alleviate heat stress in plants. The WRKYs are a large family of transcription factors that modulate many plant physiological processes, such as growth, development and responses to abiotic and biotic stresses (Chen et al., 2017a). Heat tolerance in plants requires WRKY33 (Li et al., 2011; Zhou et al., 2014). Silencing of *WRKY33* genes compromised heat tolerance and reduced heat-induced *ATG* gene expression and autophagosome formation (Zhou et al., 2014). Like COST1, however, the silencing of transcription factors, such as the WRKY family, can also have detrimental effects on agronomic performance.

Recent studies show that the transcription factor, HY5 (elongated hypocotyl 5), regulates plant autophagy in response to light-to-dark conversion and nitrogen starvation in plants. Under nitrogen-sufficient or light conditions, HY5 interacts with and recruits HDA9 (histone deacetylase 9) to *ATG5* and *ATG8e* to repress the gene expression by deacetylation of the Lys9 and Lys27 of histone 3 and inhibit autophagy. Conversely, upon dark conditions or nitrogen starvation, HY5 undergoes 26S proteasome-mediated degradation leading to dissociation of HDA9 from their target genes, thereby resulting in enhanced acetylation levels and upregulated expression of the *ATGs* and autophagy (Yang et al., 2020). Furthermore, overexpression of the transcription factor, *TGA9* (TGACG (TGA) motif-binding protein 9) transcriptionally upregulates expression of autophagy genes to activate autophagy under stress conditions. Thus, *TGA9* acts as a positive regulator of autophagy and is a possible target for improving plant stress tolerance (Wang et al., 2020).

Recently, more transcription factors have been identified in plants which can be potentially used to manipulate the autophagy pathway for stress tolerance. Brassinosteroids (BRs) regulate plant growth, development and stress responses by activating the core transcription factor BRI1-EMS-SUPPRESSOR1 (BES1), whose degradation occurs through the proteasome and autophagy pathways (Nolan et al., 2017; Wang et al., 2021). BES1 is targeted for autophagy-mediated degradation by direct interaction with ubiquitin-binding receptor protein DOMINANT SUPPRESSOR OF KAR 2 (DSK2) and is targeted to the autophagy pathway during stress *via* the interaction of DSK2 with ATG8 (Nolan et al., 2017). Recently, it has been found that ubiquitin ligase BES1-ASSOCIATED F-BOX 1 (BAF1) interacts with BES1 and mediates its ubiquitination and degradation *via* selective autophagy (Wang et al., 2021).

For instance, the plant-unique COST1 (Constitutively Stressed 1) protein negatively regulates plant drought tolerance by directly interacting with the autophagy receptor protein ATG8. Even though the loss of *COST1* improves drought tolerance by activating autophagy, it also detrimentally affects plant growth and development (Bao et al., 2020). Therefore, COST1, *via* its role in autophagy regulation, is a vital mediator that helps control the balance between growth, development and stress responses in plants (Bao, 2020; Bao et al., 2020). The high conservation of COST proteins throughout the plant kingdom indicates its potential as a gene target to enhance drought tolerance in crops (Bao et al., 2020; Bao and Bassham, 2020). Dehydrin, CAS31 (cold acclimation-specific 31) in *Medicago truncatula*, is a positive regulator of drought responses and plays a crucial role in autophagic degradation (Li et al., 2020). Recent studies show that aquaporin, PIP2;7, functions as a negative regulator of drought response (Li et al., 2020). CAS31 participates in drought-induced autophagic degradation as a cargo receptor and facilitates the autophagic degradation of PIP2; 7 and reduced root hydraulic conductivity, thus reducing water loss and improving drought tolerance (Li et al., 2020). Therefore, it is also a potential target for manipulation to improve crop tolerance.

### Manipulation of Autophagy to Improve Nutrient Remobilisation

Autophagy affects plant metabolism and is involved in efficient nutrient remobilisation from leaves to developing seeds (Marshall and Vierstra, 2018). Therefore, the manipulation of autophagy for improved nutrient recycling presents an intriguing strategy to increase the yield of seed-bearing food and biofuel crops, especially under unfavourable growth conditions or in situations where soil fertilisation is cost-prohibitive or ecologically unsound. The suppression of senescence can improve stress tolerance and yield. Sorghum stay-green hybrid cultivars have significant yield advantages under postanthesis drought compared to senescent hybrid lines (Borrell et al., 2000a,b). In addition to increasing yield potential, nutrient recycling may lower the requirement of chemical inputs, providing both environmental and economic benefits. The manipulation of the metabolome *via* the targeted degradation of specific constituents can also increase agronomic productivity in crop species (Marshall and Vierstra, 2018). Studies show that transcription factors, such as Hsf1A, an essential component of plant drought responses, activate *ATG* expression upon stress conditions and are potential gene targets for crop improvement (Wang et al., 2015).

Plants containing mutated ATG8-ATG12 conjugation pathways display accelerated leaf senescence and hypersensitivity to carbon and nitrogen limitation (Doelling et al., 2002; Yoshimoto et al., 2004; Hofius et al., 2009; Chung et al., 2010). Conversely, the overexpression of *ATG* (autophagy stimulation) enhances plant growth, fitness, seed set and stress tolerance (Xia et al., 2012; Wang et al., 2016; Minina et al., 2018; Sun et al., 2018c). Another potential strategy for the suppression of senescence and improved stress tolerance is the overexpression of *KIN 10*, the catalytic subunit of *SnRK1*. Expression of *KIN10* promotes autophagy, delays senescence and increase tolerance to nutrient starvation conditions (Chen et al., 2017b; Soto-Burgos and Bassham, 2017; Huang et al., 2019).

Autophagy is a strong determinant of seed quality and a critical component of the micronutrient seed filling (Pottier et al., 2019). *Arabidopsis thaliana* autophagy mutants are inefficient at translocating iron from vegetative organs to the seeds (Pottier et al., 2019). Plants also use senescent vegetative organs to sequester manganese and zinc into seeds. The lower amounts of zinc and manganese in the seeds of autophagy mutants suggest that their translocation depends on autophagy (Pottier et al., 2019). Future work should investigate the potential manipulation of autophagy in crops at the seed filling stage to increase the pool of nutrients available for subsequent translocation to seeds.

Recently, researchers demonstrated an essential role for the autophagy-related kinase, PI3K, in symbiotic interactions between common bean plants and rhizobia bacteria or arbuscular mycorrhizal fungi (Estrada-Navarrete et al., 2016). Transgenic PI3K-RNAi plants display decreased root hair growth and curling (Estrada-Navarrete et al., 2016). Additionally, infection thread growth, nodule number and symbiosome formation were severely affected (Estrada-Navarrete et al., 2016). However, more work is required to conclude that autophagy is directly involved in symbiotic interactions because the PI3K complex is involved in many other biological processes (Tang and Bassham, 2018). Manipulation of autophagy pathways could potentially enhance symbiotic interactions with legumes and subsequently reduce fertiliser inputs (Blair et al., 2016).

### Manipulation of Autophagy to Modulate Plant Development

Autophagy is highly involved in plant development, pollen maturation, lipid metabolism and nutrient supply in anthers. It is crucial in angiosperm sexual reproductive development and is potentially involved in the degradation of intracellular components, such as plastids and lipid bodies and the regulation of lipid metabolism during pollen maturation (Hanamata et al., 2014; Kurusu et al., 2014; Kurusu and Kuchitsu, 2017).

During Arabidopsis seed maturation, transcript levels of almost all autophagy-related genes increase in the silique (Di Berardino et al., 2018). Similarly, maize developing endosperms contain high amounts of lipidated *ATG8* (ATG8-PE; Chung et al., 2009). Furthermore, transcriptome analyses show increased ATG transcript accumulation in the endosperm, but not the embryo, suggesting that autophagy participates in the endosperm’s maturation and death during seed development (Li et al., 2015a). The manipulation of autophagy pathways may improve seed vigour and storage.

Photoperiod regulates autophagy, and thus, autophagy may be an intriguing target for the development of determinant crops or crops with shortened or prolonged lifecycles. Day length and autophagy determine the number of fertile florets, and developmentally generated sugar starvation triggers floret autophagy in wheat (Ghiglione et al., 2008). Long days intensify this process due to the increased carbohydrate consumption caused by accelerated plant development (Ghiglione et al., 2008). It is feasible to manipulation flowering time by either activating or suppressing autophagy pathways. The activation of autophagy delays senescence and flowering, while plants with dysfunctional autophagy pathways display accelerated senescence.

Collectively, these findings display substantial potential for manipulating autophagy as a target for the development of more resilient crops that can withstand future inclement environmental conditions.

### Pharmacological Elicitation of Plant Autophagy Pathways

Compared with animals, the pharmacological manipulation of autophagy with stimulants, such as rapamycin, fumonisin B1, tunicamycin, polyamines and suppressors wortmannin concanamycin A in agricultural applications, remains untested (Avin-Wittenberg et al., 2018). It is infeasible to spray agricultural fields with these chemicals; however, the use of nanoparticle carriers to deliver such autophagy regulators may be possible, and researchers have trialled such approaches in human cells (Chen et al., 2018; Fu et al., 2018) by inducing responses that prepare the plants for future more severe stresses (Signorelli et al., 2019). There is a potential to use nanoparticles and encapsulated autophagy modulators to deliver mild stress inducers, such as H_2_O_2_ and NaCl, or autophagy elicitors, for priming autophagy to prevent susceptibility of crop plants to abiotic stresses. Using chemical elicitors to manipulate autophagy pathways has some disadvantages, such as off-target effects, drug instability and cell permeability (Avin-Wittenberg et al., 2018). However, this approach has some benefits too.

Before using chemical autophagy modulators for agricultural benefits, it is important to investigate their specificity. Recently, Dauphinee *et al* designed a novel four phase pipeline to identify autophagy-modulating chemicals (Dauphinee et al., 2019). In addition to identifying novel chemical regulators, the pipeline provides in-depth mechanistic understanding of the modulator’s activity and information for optimisation of specificity and potency relevant for agricultural applications (Dauphinee et al., 2019).

The use of chemical modulators of autophagy does not require extensive plant breeding. It also circumvents many of the regulatory and public perception hurdles observed with genetically modified (GM) crops (Avin-Wittenberg et al., 2018).

## Is selective autophagy as a promising strategy to improve crop fitness under stress?

In contrast to bulk degradation, autophagy can be targeted to identify and engulfment particular organelles, protein complexes, protein aggregates or pathogens into autophagosomes (Izumi et al., 2017; Wang et al., 2017; Marshall and Vierstra, 2018). This form of autophagy is termed selective autophagy and is defined as autophagy that requires additional autophagic receptors (e.g., NBR1) that help deliver specific cellular cargos (damaged, degraded, misfolded proteins and organelles) to the autophagosome for degradation in the vacuole (Svenning et al., 2014; Michaeli et al., 2016). The combination of bulk degradation with selectivity enables autophagy to mediate precise and context-dependent responses intricately. For example, a healthy cellular mitochondrial population is key to maintaining energy levels. However, misfunctioning mitochondria are a significant ROS source that directly damages cellular components and potentially trigger cell death. As mentioned, the accumulation of ROS triggers autophagy. Suppose this is not sufficient to protect the cell. In that case, selective autophagy pathways are activated that degrade dysfunctional mitochondria (the ROS source), termed ‘Mitophagy’, to mitigate stress and facilitate homeostasis (Kanki et al., 2015; Broda et al., 2018; Ma et al., 2021; Nakamura et al., 2021a,b; Ren et al., 2021). In contrast, bulk turnover of cytoplasmic components occurs in cells during development to remove cell debris and to replenish needed pools of amino acids, sugars, fatty acids and nucleotides during nutrient deprivation; it typically involves the random uptake of cytoplasm into the phagophore (Marshall and Vierstra, 2018; Signorelli et al., 2019). Combining bulk and selective autophagy may provide the versatility required for plants to respond to various stresses of different severities and duration.

Identification of selective autophagy in plants provided insight into how plants target unwanted or damaged organelles for degradation as a housekeeping mechanism and promote nutrient recycling required for plant growth and development. It also revealed new tools for innate immunity by which cargo receptors help eliminate invading pathogens. Selectivity in autophagy is conferred by cargo receptor proteins, which are able to simultaneously interact with the cargo and ATG8 family proteins on the autophagosomal membrane (Zaffagnini and Martens, 2016). In addition to interaction *via* the ATG8-interacting motif (AIM), recent research has identified an alternative site that promotes interaction between the cargo receptor and ATG8 *via* a new binding site that exploits ubiquitin-interacting motif (UIM)-like sequences, which interacts with the UIM-docking site (UDS; Marshall et al., 2019). This discovery expands on the complexity of the autophagy system and the pathways involved in selective cargo recruitment. The UDS targets non-functional CDC48/p97 complexes to the ATG8 decorated autophagosomes. CDC48/p97 turnover is vital in eukaryotes because it is essential for endoplasmic reticulum-associated degradation (ERAD) and other protein quality control pathways related to several human diseases (Ye et al., 2001; Tang and Xia, 2016). Therefore, the UDS interface is critical for maintaining eukaryotic proteostasis (Marshall et al., 2019). Several autophagy receptors are present in plants and are extensively reviewed in the literature (Marshall and Vierstra, 2018; Avin-Wittenberg, 2019). The knowledge of selective autophagy mechanisms can help guide the manipulation of autophagy to develop robust, high yielding crops.

## Conclusion and Future Directions

Over the past decade, autophagy research has expanded from model plants to crop species. The roles of autophagy in stress response, plant microbial interactions and development are established in multiple plant species. Recently, selective autophagy for the targeted degradation of organelles or protein complexes has gained significant attention. The manipulation of autophagy pathways shows excellent promise for the improvement of crop productivity under challenging environmental conditions. To harness the full potential of selective autophagy in agriculture, however, it is essential to identify and characterise the adapters/receptor proteins or other new compounds that help mediate the process in plants and the signals that act in specific cargo recruitment. We still need clarification on species-specific differences in selective autophagy pathways in crop plants and their responses to stress conditions. This information could elude to essential factors of stress perception and adaptation in plants. The manipulation of autophagy can improve environmental stress tolerance, nutrient remobilisation (i.e., growth and yield), pathogen resistance in the field and mitigate post-harvest losses. Identifying the regulatory mechanics of plant autophagy presents unique and exciting opportunities for plant biologists and agricultural scientists to understand how plant cells perceive stress and improve yield potential and stress tolerance in future predicted inclement environments.

## Author Contributions

NT and BW contributed to conception, design, and drafting the manuscript. SB, BF, MK, SM, and BW contributed to the critical revision of the article. All authors contributed to the article and approved the submitted version.

## Funding

This work was supported by Queensland University of Technology (QUT) scholarship, and an Advance QLD Research Fellowship package AQRF14816-17RD2, AQRF04016-17RD2, and AQRF14716-17RD2.

## Conflict of Interest

The authors declare that the research was conducted in the absence of any commercial or financial relationships that could be construed as a potential conflict of interest.

## Publisher’s Note

All claims expressed in this article are solely those of the authors and do not necessarily represent those of their affiliated organizations, or those of the publisher, the editors and the reviewers. Any product that may be evaluated in this article, or claim that may be made by its manufacturer, is not guaranteed or endorsed by the publisher.
